# Murine Mesenchymal Stem Cells Exhibit a Restricted Repertoire of Functional Chemokine Receptors: Comparison with Human

**DOI:** 10.1371/journal.pone.0002934

**Published:** 2008-08-13

**Authors:** Giselle Chamberlain, Karina Wright, Antal Rot, Brian Ashton, Jim Middleton

**Affiliations:** 1 Leopold Muller Arthritis Research Centre, Medical School, Keele University, RJAH Orthopaedic Hospital, Oswestry, Shropshire, United Kingdom; 2 Spinal Studies, Medical School, Keele University, RJAH Orthopaedic Hospital, Oswestry, Shropshire, United Kingdom; 3 Novartis Institutes for BioMedical Research, Vienna, Austria; Institut Pasteur Korea, Republic of Korea

## Abstract

Mesenchymal stem cells (MSCs) are non-haematopoeitic, stromal cells that are capable of differentiating into mesenchymal tissues such as bone and cartilage. They are rare in bone marrow, but have the ability to expand many-fold in culture, and retain their growth and multi-lineage potential. The properties of MSCs make them ideal candidates for tissue engineering. It has been shown that MSCs, when transplanted systemically, can home to sites of injury, suggesting that MSCs possess migratory capacity; however, mechanisms underlying migration of these cells remain unclear. Chemokine receptors and their ligands play an important role in tissue-specific homing of leukocytes. Here we define the cell surface chemokine receptor repertoire of murine MSCs from bone marrow, with a view to determining their migratory activity. We also define the chemokine receptor repertoire of human MSCs from bone marrow as a comparison. We isolated murine MSCs from the long bones of Balb/c mice by density gradient centrifugation and adherent cell culture. Human MSCs were isolated from the bone marrow of patients undergoing hip replacement by density gradient centrifugation and adherent cell culture. The expression of chemokine receptors on the surface of MSCs was studied using flow cytometry. Primary murine MSCs expressed CCR6, CCR9, CXCR3 and CXCR6 on a large proportion of cells (73±11%, 44±25%, 55±18% and 96±2% respectively). Chemotaxis assays were used to verify functionality of these chemokine receptors. We have also demonstrated expression of these receptors on human MSCs, revealing some similarity in chemokine receptor expression between the two species. Consequently, these murine MSCs would be a useful model to further study the role of chemokine receptors in *in vivo* models of disease and injury, for example in recruitment of MSCs to inflamed tissues for repair or immunosupression.

## Introduction

Mesenchymal stem cells (MSCs) are non-haematopoeitic, stromal cells that are capable of differentiating into, and contribute to the regeneration of, mesenchymal tissues such as bone, cartilage, muscle, ligament, tendon, adipose and stroma [1], [2]. It has also been documented that MSCs can express cardio-myogenic phenotypes [3] as well as being able to differentiate into neural elements *in vitro* [4]. MSCs have the ability to expand many-fold in culture, whilst retaining their growth and multilineage potential. They are also reported to have immunosuppressive properties [5] and are regarded as non-immunogenic, therefore transplantation into an allogenic host may not require immunosupression [6].

These properties of MSCs make the cells ideal candidates for tissue engineering, and cellular and gene therapy. It has been shown that MSCs, when transplanted systemically, are able to migrate into damaged or diseased tissues [7], [8], such as ischemic brain [9], [10], infarcted myocardium [11] and injured lung [12], where they can show clinical benefit. These findings suggest that MSCs possess migratory capacity; however the mechanisms underlying the migration of these cells remain unclear.

Chemokine receptors and their ligands, and adhesion molecules play an important role in tissue-specific homing of leukocytes [13], and have also been implicated in trafficking of haematopoietic precursors into and through tissue [14]. Chemokines presented on endothelial cells trigger integrin activation and arrest of those leukocytes that carry the corresponding receptors [15]. The blood vessel at which a leukocyte undergoes extravasation is tightly controlled by the range of chemokine receptors and adhesion molecules expressed on the leukocyte cell surface, often referred to as the cell's address code [16].

Several studies have reported the functional expression of various chemokine receptors on human MSCs [17]–[22]; some of the results are inconsistent between research groups, and many studies have not looked at the full panel of chemokine receptors. Various adhesion molecules are also known to be expressed on human MSCs [23], [24], some of which may be functionally important in the adhesion of MSCs to the endothelium [25]. However, little is known about the mechanism of MSC transendothelial migration, and which chemokine receptors may be involved [7], [8].

To our knowledge, no one has reported which chemokine receptors are functionally expressed on murine bone marrow derived MSCs, and compared them to those expressed by human MSCs. In this study, we have demonstrated the functional presence of chemokine receptors on murine MSCs, and have shown that their expression profile exhibits similarities to that of human MSCs. This information can be used to further study the mechanism of transmigration of MSCs into tissues, specifically in a mouse model, and may allow the development of therapeutic strategies to enhance the recruitment of ex-vivo cultured MSCs to damaged or diseased tissues.

## Results

**Figure 1:**
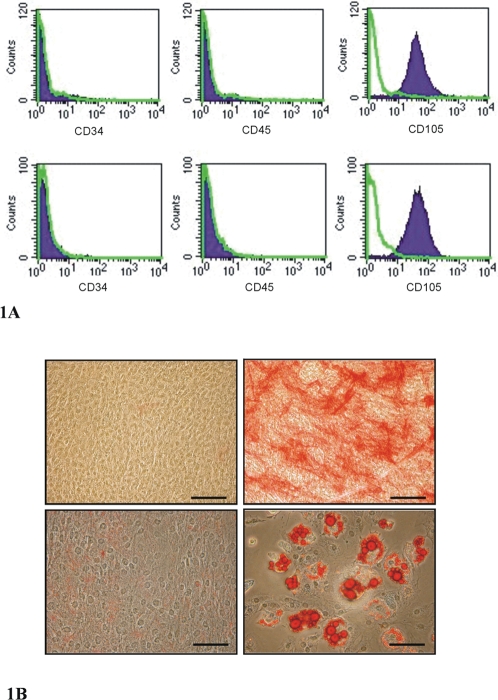
Characterisation of primary MSCs by flow cytometry and differentiation assays. A. Flow cytometry analysis of all primary murine MSC cultures (upper panel) and human MSC (lower panel) cultures showed they were CD34 and CD45 negative, and CD105 positive. CD molecule antibody staining is represented by the filled histogram; isotype control staining is represented by the green line. B. Murine MSCs (CD45^−^, CD34^−^, CD105^+^) incubated in osteogenic medium for 21 days stained positive for alkaline phosphatase activity (top right), whereas murine MSCs incubated in culture medium alone did not stain positive (top left). Murine MSCs incubated in adipogenic medium for 21 days showed fat droplets in the cells stained with Oil Red O (bottom right), whereas murine MSCs incubated in culture medium alone showed no positive staining (bottom left). The black bar represents 200 µm in the top panels and 100 µm in the bottom two panels.

Assays were performed on primary murine cells from passage 7–9. All primary murine MSC cultures were shown to be CD34^−^, CD45^−^, and CD105^+^ (Figure 1A), as well as demonstrating osteogenic and adipogenic differentiation potential (Figure 1B). The murine MSC cell line C3H/10T1/2 has already been shown to differentiate down the osteogenic, adipogenic and chondrogenic pathways [26] as well as having the same immunosuppressive properties as bone marrow-derived primary human MSCs [27]. All human MSC cultures were shown to be CD34^−^, CD45^−^, and CD105^+^ (Figure 1A), and were used between passages 2 and 5.

**Figure 2:**
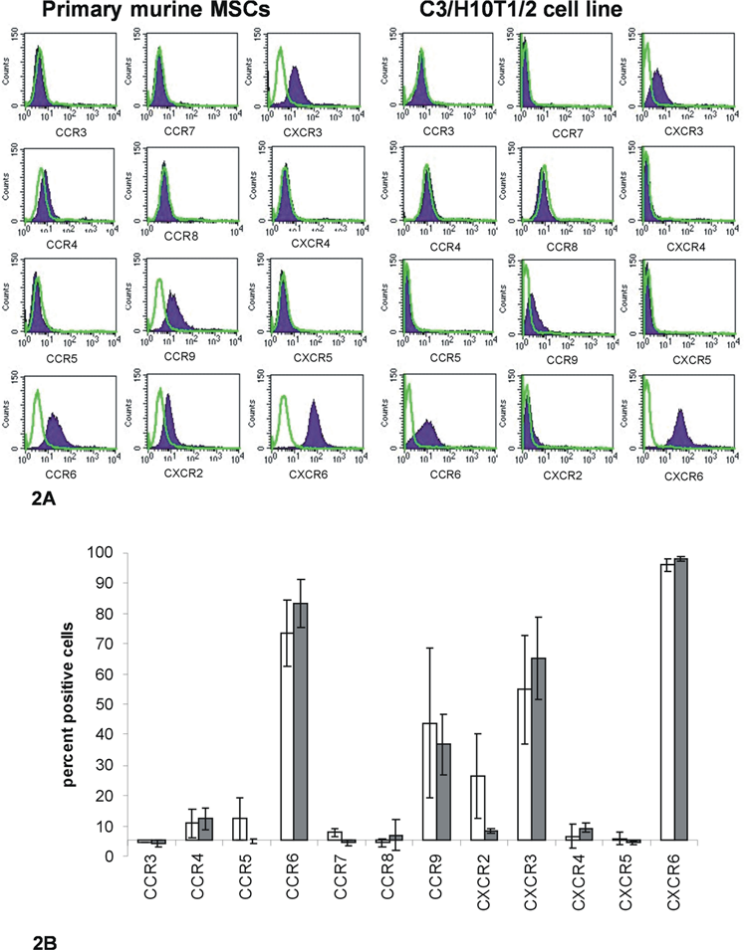
Analysis of chemokine receptor expression on murine MSCs. A Flow cytometry analysis of chemokine receptor expression on the surface of primary murine MSCs and the C3H/10T1/2 cell line. Chemokine receptor antibody staining is represented by the filled histogram; isotype control staining is represented by the green line. Histograms are representative of three independent experiments, performed at three different passages. B Mean percentage positive MSCs is shown for each receptor (±SE, n = 3) detected by flow cytometry on the surface of primary murine MSCs (white bars) and the C3H/10T1/2 cell line (grey bars).

**Figure 3:**
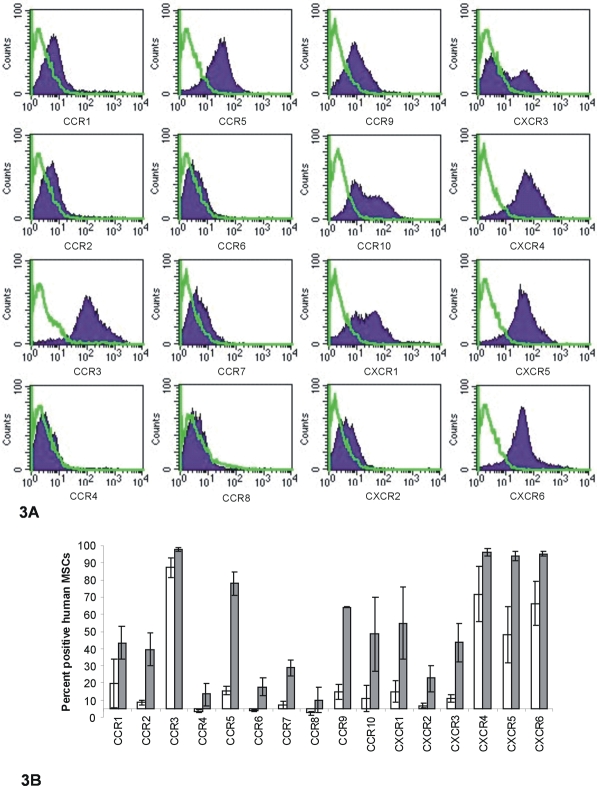
Analysis of chemokine receptor expression on human MSCs. A Flow cytometry analysis of chemokine receptor expression on the cell surface of human MSCs. Chemokine receptor antibody staining is represented by the filled histogram; isotype control staining is represented by the green line. Histograms are representative of three different patients' cells, all removed from flasks by EDTA alone. B Mean percentage positive MSCs is shown for each receptor (±SE, n = 3) detected by flow cytometry on the surface of human MSCs removed from the flask by trypsin-EDTA (white bars), or by EDTA alone (grey bars).

The cell surface expression of chemokine receptors (CCR3-9 and CXCR2-6) was assessed in primary murine MSC cultures at passages 7–9 by flow cytometry (Figure 2) as described. C3H/10T1/2 cells were also assessed for chemokine receptor expression (Figure 2) at passage 12–14. We could not examine expression of CCR1, CCR2 or CXCR1 as antibodies to the murine form of these molecules could not be found. The cell surface expression of chemokine receptors (CCR1-10 and CXCR1-6) was also assessed in primary human MSC cultures by flow cytometry, however, it was found that the trypsin used to remove cells from the flask was removing a large proportion of chemokine receptors from the surface of the cells. Therefore chemokine receptor expression was assessed by flow cytometry in three more patients' MSC cultures comparing the use of trypsin-EDTA to remove cells or EDTA alone (Figure 3). With EDTA alone a greatly increased number of human MSCs showed expression of all the chemokine receptors tested. This was not a problem with the murine cells as these came off the flasks within 30 seconds using trypsin, and furthermore, using EDTA alone did not show significantly higher expression of chemokine receptors than with trypsin (data not shown). Cells were considered to be positive for a marker if the fluorescence intensity was higher than 95% of the cells stained with the relevant isotype control.

A high percentage of both the primary murine MSCs and the C3H/10T1/2 cells were shown to express CXCR6 on the cell surface (96±2% and 98±1% respectively) (Figure 2); interestingly, this chemokine receptor was also expressed on a high proportion (95±1%) of human bone-marrow-derived MSCs (Figure 3). The receptors CCR6, CXCR3 and CCR9 were also expressed on the cell surface of a high proportion of both the primary murine MSCs (73±11%, 55±18% and 44±25% respectively) and C3H/10T1/2 cells (83±8%, 65±14% and 37±10% respectively) (Figure 2). A smaller proportion of both murine cell types were also shown to express CXCR2 (26±14% of primary cells, 8±1% of C3H/10T1/2 cells). We also detected expression of these receptors on human MSCs by flow cytometry (Fogure 3): CCR6 (on 20±8% of cells), CXCR3 (on 54±8% of cells), CCR9 (64±1% of cells) and CXCR2 (on 30±1% of cells). We detected expression of every other receptor bar CCR3, but only on a negligible proportion of murine cells (Figure 2), whereas some of these receptors were expressed on a large proportion of the human MSCs (CCR3 (98±1%), CCR5 (78±7%), CCR7 (29±4%), CXCR4 (96±2%), and CXCR5 (94±3%)), as well as those receptors not tested for on the murine cells (CCR1 (43±10%), CCR2 (40±10%), CCR10 (48±22%) and CXCR1(55±21%)).

**Figure 4:**
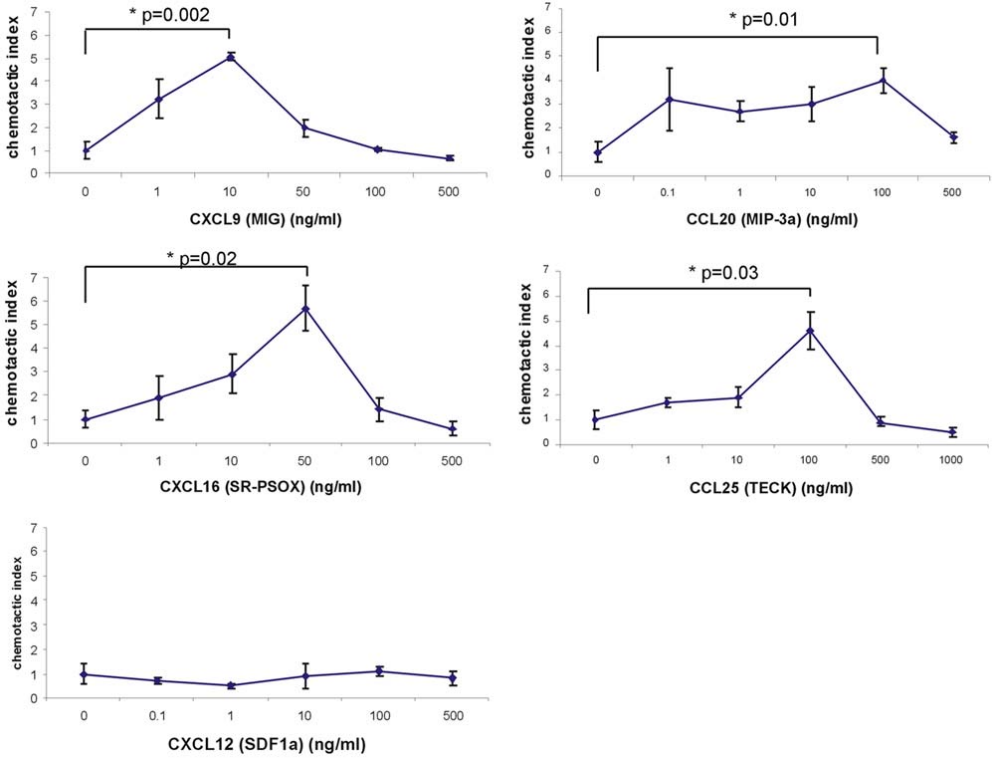
Analysis of murine chemokine receptor function by chemotaxis. Chemotaxis assays were performed on murine MSCs at passage 7. Each chemokine concentration was performed in triplicate per assay, and each assay was repeated three times. Results are expressed as the mean number of migrated cells over control cells (basal migration without chemotactic stimulus) (mean±SE; n = 3), counted in five microscope fields of view at x130 magnification. *Denotes significant difference compared to negative control with no chemokine. The mean number of cells that migrated in the absence of chemokine, per field of view, was 12.1±3.2.

Next, the response of the CXCR6, CXCR3, CCR6 and CCR9 receptors to their corresponding ligands (CXCL16 (SR-PSOX), CXCL9 (MIG), CCL20 (MIP-3α/LARC) and CCL25 (TECK) respectively) was tested on the murine MSCs using chemotaxis assays (Figure 4) as a large proportion of the cells expressed these receptors. As the murine MSCs showed little or no cell-surface expression of CXCR4 by flow cytometry, the chemokine CXCL12 (SDF-1α) (the ligand for CXCR4) was also used as a negative control. The chemokines CXCL16, CXCL9, CCL20 and CCL25 all induced significant migration of murine MSCs in a dose-dependent manner compared to media alone (Figure 4), whereas CXCL12 did not induce migration, indicating that the receptors detected on these cells by flow cytometry are functional.

## Discussion

Many recent studies have reported the functional expression of various different chemokine receptors on human MSCs [17]–[22]; however, results have been contradictory in many cases. We have shown here that the reason for this may be due in part to the use of differing concentrations of trypsin, for various lengths of time, to remove cells from the tissue culture flask (Figure 3B). Human MSCs show a vast increase in the proportion of cells expressing chemokine receptors on their surface when removed with EDTA alone rather than trypsin, indicating the sensitivity of some or possibly all chemokine receptors to trypsin digestion. We did not see this with the murine cells as the trypsin was left on the cells for such a short time, however, we would not recommend the use of trypsin to remove any species' cells in this type of study as chemokine receptors and possibly many other molecules expressed on the cell surface are sensitive to trypsin digestion. The current investigation showed abundant expression of CXCR4 on human MSCs in agreement with several other studies [17], [19]–[21] who also showed chemotactic responses to CXCL12. However others have reported little or no expression of this receptor on human MSCs [18], [22]. This may have been due to variable use of trypsin or other experimental conditions.

We have demonstrated functional expression of CCR6, CCR9, CXCR3 and CXCR6 on a large proportion of murine MSCs and interestingly, shown expression of all four of these receptors on a proportion of human MSCs. Other groups have also demonstrated functional expression of one or more of these receptors on human MSCs [17]–[21].

All four of these receptors (CCR6, CCR9, CXCR3 and CXCR6) have been shown to be involved in recruitment of immune cells to areas of inflammation. CCR6 is involved in mucosal humoral immunity and intestinal T cell homing [28], and it has recently been reported that Th17 cells expressing CCR6 are preferentially recruited to inflamed joints via its ligand CCL20 in an animal model of rheumatoid arthritis [29]. In fact, both CXCR3 and CXCR6 have also been implicated in the recruitment of T cells to inflamed tissues in autoimmune arthritis [30], [31], as well as other inflammatory conditions. CCR9 is known to be involved in homing of T cells and plasma cells to the intestine [28], and plays a role in inflammatory diseases of the gut such as Crohn's disease [32]. Considering the known functions of these receptors in relation to recruitment and homing of immune cells to inflamed tissues, it is reasonable to hypothesize that these receptors may also be involved in the recruitment and homing of murine and human MSCs to inflamed tissues, either for the purpose of tissue regeneration or in an immunosuppressive capacity.

Some differences were apparent between the spectra of chemokine receptors expressed by human and murine MSCs. CCR3, CCR5, CXCR4 and CXCR5 were present abundantly on human cells whereas low levels of these receptors occurred on murine cells. Therefore in instances where these receptors are shown to be important in the recruitment of MSCs in humans, murine MSCs may not provide a useful model.

We have reported that murine MSCs demonstrate selective expression of functional chemokine receptors, with similarities to human MSCs as well as differences. Thus these murine MSCs would be a useful model to further study the role of particular chemokine receptors in *in vivo* models of disease and injury, for example in recruitment of MSCs to inflamed tissues.

## Materials and Methods

### Isolation and Expansion of Murine MSCs

Primary murine MSCs were obtained from BALB/c mice, 6–10 weeks old. MSCs were isolated as previously described [33]. Briefly, marrow was removed from the long bones and cells plated out in cell isolation media (CIM) (RPMI-1640 (Lonza, UK) with 9% FBS, 9% horse serum (both Gibco, UK)) at 37°C, 5% CO_2_. After 24 hours, non-adherent cells were removed. After 4 weeks cells were re-plated at 100 cells per cm^2^ in complete expansion media (CEM) (Iscove Modified Dulbecco Medium (Lonza) with 9% FBS, 9% horse serum) to expand MSCs.

The murine cell line C3H/10T1/2 was purchased from LGC Promochem, a distributor for the American Tissue Culture Collection. Cells were cultured in Basal Medium Eagles (Lonza) with 10% FBS.

### Isolation and Expansion of Human MSCs

Human MSCs were obtained from patients with osteoarthritis undergoing total hip replacement, as described previously [25]. Briefly, marrow from femoral head cancellous bone was obtained from osteoarthritis patients after informed consent. The mononuclear cell fraction was prepared by density gradient centrifugation, and then seeded at a density of approximately 20×10^6^ cells per T75 flask, in DMEM-F12 medium (Lonza,) with 10% FBS. After 24 hours incubation at 37°C, 5% CO_2_, non-adherent cells were removed and the remaining cells were cultured until reaching 70–80% confluence. Cells were then passaged and then re-plated at a density of approximately 2×10^3^ cells per cm^2^ for further expansion.

### Flow Cytometric Analysis

Murine and human MSCs were analysed for membrane receptor expression using a three-step labeling procedure. Cells were incubated at 4°C for 30 minutes with the relevant primary anti-mouse or anti-human antibodies, then after washing, cells were subsequently incubated with a biotinylated anti-rat Ig, anti-mouse Ig, anti-rabbit Ig or anti-goat Ig antibody, and then with Streptavidin-PE conjugate. As a negative control, cells were incubated with the same species isotype controls as the primary antibodies. A minimum of 10,000 events were recorded for each analysis, using a FACScan flow cytometer and analysed using cell quest software (BD Biosciences, UK).

Antibodies used in this study were as follows: anti-human CCR1 (used at 1 in 100 dilution), CCR2 (1 in 200), CCR3 (1 in 100), CCR5 (1 in 200), CCR6 (1 in 200), CCR7 (1 in 200), CCR8 (1 in 20), CCR9 (1 in 20), CXCR1 (1 in 100), CXCR2 (1 in 100), CXCR3 (1 in 200), CXCR4 (1 in 50), CXCR5 (1 in 50), and CXCR6 (1 in 50), anti-mouse CCR6 (1 in 50), CCR9 (1 in 50), CXCR2 (1 in 50), CXCR3 (1 in 50), and CXCR6 (1 in 50) and anti-mouse CD105 (all from R&D Systems, UK), anti-human CCR4 (1 in 100), anti-mouse CCR3 (1 in 50), CCR5 (1 in 50), CXCR4 (1 in 50), CXCR5 (1 in 50) and CD45 PE/Cy5 (all from BD Pharmingen, UK), anti-human CCR10 and anti-mouse CCR4 (1 in 50) and CCR8 (1 in 50) (Abcam, UK), anti-human CD105 FITC (1 in 50), CD34 PE (1 in 100), CD45 PE/Cy5 (1 in 100) and anti-mouse CD34 PE (1 in 100) (Immunotools, Germany), and anti-mouse CCR7 (BioLegend, UK).

Secondary and negative control and blocking antibodies used in this study were: goat IgG, rat IgG2_a_, rat IgG2_b,_ rat IgG2a PE (all from R&D Systems, UK), mouse IgG1, mouse IgG2_a_, mouse IgG2_b,_ and rabbit Ig (all from DakoCytomation, Denmark), streptavidin-PE conjugate (1 in 200), biotin anti-rat Ig (1 in 200), biotin anti-mouse Ig (1 in 200), rat IgG_2c_, rat IgG_2b_ PE, mouse FC block (all from BD Pharmingen, UK), mouse IgG1 PE, mouse IgG2a PE/Cy5, mouse IgG2a FITC (all from Immunotools, Germany), biotin anti-goat IgG (1 in 200) and biotin anti-rabbit IgG (1 in 200) (both from Abcam, UK).

### Differentiation Assays

For osteogenic differentiation, murine MSCs were incubated in CEM with ascorbate-2-phosphate (88 ng/ml), dexamethasone (10^−8^M, Sigma-Aldrich, UK) and β-glycerophosphate (10mM, Sigma-Aldrich). For adipogenic differentiation, mMSCs were incubated in CEM with ITS (Insulin, Transferrin, Selenium)+Premix (Gibco, UK), dexamethasone (10^−6^M), 3-isobutyl-1-methylxanthine (0.5 µM, Sigma-Aldrich) and indomethacin (100 µM, Sigma-Aldrich). After three weeks, cells were fixed and stained with Fast Red TR/napthol (Sigma-Aldrich) for alkaline phosphatase activity (osteoblastic differentiation), or with Oil Red-O for adipogenic differentiation.

### Migration Assays

Murine MSC chemotaxis was determined in a 48-well modified Boyden chamber (Neuroprobe, Receptor Technologies, UK). Serial dilutions of chemokine in FCS-free media were placed in the lower wells. A polycarbonate membrane with 12 µm pores (Neuroprobe) was used, and MSCs (10^5^ cells/ml) were placed in the upper wells, and incubated at 37°C for 4 hours. Filters were removed, wiped off on the upper side, air-dried and stained with haemacolor (Merck, UK). Migrated MSCs were counted in 5 fields of view (x130 magnification). Where appropriate, two-tailed T-tests were performed on cell counts at optimum chemokine concentration for chemotaxis, to determine significance as compared to no chemokine.
